# A randomized controlled trial of stem cell injection for tendon tear

**DOI:** 10.1038/s41598-021-04656-z

**Published:** 2022-01-17

**Authors:** Se-Woong Chun, Won Kim, Sang Yoon Lee, Chai-Young Lim, Keewon Kim, Jeong-Gil Kim, Chul-Hyun Park, Sung Hwan Hong, Hye Jin Yoo, Sun G. Chung

**Affiliations:** 1 Department of Rehabilitation Medicine, Gyeongsang National University Changwon Hospital, Gyeongsang National University College of Medicine, Changwon, Gyeongsangnam-do Republic of Korea; 2 Department of Rehabilitation Medicine, Asan Medical Center, University of Ulsan College of Medicine, Seoul, Republic of Korea; 3 Department of Rehabilitation Medicine, College of Medicine, Seoul National University, 101 Daehak‐ro, Jongno‐gu, Seoul, 110‐744 Republic of Korea; 4 Department of Rehabilitation Medicine, Seoul National University College of Medicine, SMG-SNU Boramae Medical Center, Seoul, Republic of Korea; 5 Present Address: Department of Rehabilitation Medicine, Seoul National University Hospital, Seoul, Republic of Korea; 6 Armed Forces Daejeon Hospital, Daejeon, Republic of Korea; 7 Department of Physical and Rehabilitation Medicine, Kangbuk Samsung Hospital, Sungkyunkwan University School of Medicine, Seoul, Republic of Korea; 8 Department of Radiology, Seoul National University College of Medicine, Seoul, Republic of Korea; 9 Department of Radiology, Seoul National University Hospital, Seoul, Republic of Korea; 10 Institute of Aging, Seoul National University, Seoul, Republic of Korea

**Keywords:** Stem cells, Medical research

## Abstract

Tendons have limited reparative ability and perform a relatively simple mechanical function via the extracellular matrix. Thus, the injured tendon might be treated successfully by stem cell transplantation. We performed a randomized, controlled study to investigate the effects of mesenchymal stem cell injection for treating partial tears in the supraspinatus tendon. We enrolled 24 patients with shoulder pain lasting more than 3 months and partial tears in the supraspinatus tendon. Participants were assigned to three groups: stem cells in fibrin glue, normal saline/fibrin glue mixture, and normal saline only, with which intra-lesional injection was performed. Pain at activity and rest, shoulder function and tear size were evaluated. For safety measures, laboratory tests were taken and adverse events were recorded at every visit. Participants were followed up at 6, 12 weeks, 6, 12 months and 2 years after injection. The primary outcome measure was the improvement in pain at activity at 3 months after injection. Twenty-three patients were included in the final analysis. Primary outcome did not differ among groups (*p* = 0.35). A mixed effect model revealed no statistically significant interactions. Only time significantly predicted the outcome measure. All participants reported transient pain at the injection site. There were no differences in post-injection pain duration or severity. Safety measures did not differ between groups, and there were no persistent adverse events. Stem cell injection into supraspinatus partial tears in patients with shoulder pain lasting more than 3 months was not more effective than control injections.

ClinicalTrials.gov Identifier: NCT02298023

## Introduction

The function of the tendon to transmit force generated by the muscle to the bone results in perpetual tension with or without compression and friction. Even mechanical loading within the physiological capacity of the tendon can damage the microstructure when applied repetitively^1^; this damage can be repaired by intrinsic healing processes orchestrated by tenoblasts^2^. However, the mature tendon is sparsely populated by cells, accounting for approximately 5% of the tissue volume, of which less than 1% possess progenitor cell properties^3^. Thus, replenishing the local cell population with stem cells to augment the regenerative potential of the tendon is appealing as a novel treatment for tendinopathy.

Amid positive reports from stem cell clinical trials for connective tissue lesions, i.e., Crohn’s anal fistula^4^ and bony fracture^5^, the efficacy of mesenchymal stem cell (MSC)-mediated tendon regeneration has been demonstrated by its structural and biomechanical effects in various animal models of tendinopathy^6,7^. Recently, a clinical trial on intra-tendon MSC injection for rotator cuff tear reported promising results^8^. The authors even observed complete resolution of the tendon tear in some participants via arthroscopy. However, the single-arm study design could not exclude the effects of natural recovery, adjunctive treatments, and the placebo effect, preventing them from making conclusions regarding the real effects of MSC injection. A comparative clinical trial is warranted to verify the clinical efficacy of MSCs in rotator cuff tears.

Therefore, in this study, we conducted the first randomized, placebo-controlled trial to investigate the effects of MSC injection into tendinous lesions. We hypothesized that delivering MSCs into tear lesions, partial tears of the supraspinatus tendon in this study, would augment the extent and rate of clinical improvement compared with delivering control injectates and that the MSC injection would be well tolerated.

## Methods

### Study overview

This randomized placebo-controlled trial was conducted from November 2014 to April 2018 at Seoul National University Hospital in Seoul, Korea. We adopted a three-arm study design including intervention, active control, and control groups. The protocol (ClinicalTrials.gov Identifier: NCT02298023, first registration: 21/11/2014) and informed consent were approved prior to the trial. The trial ended as scheduled after recruiting the target population. Written informed consent was obtained from all participants prior to the enroll. The trial was conducted according to the principles in the Declaration of Helsinki and followed the Good Clinical Practice guidelines.

### Participants

#### Screening, enrollment, and random allocation

Volunteers were screened by a physiatrist subspecializing in musculoskeletal rehabilitation (L.CY, C.SW) with at least 5 years of clinical practice and experience in ultrasonographic examination. The screening included taking the history of shoulder pain, examining the range of motion of the shoulder, performing empty can tests, and evaluating ultrasonographic findings. Informed consent was obtained from patients who had a relevant history and compatible findings in physical/ultrasonographic examinations. Subsequent magnetic resonance imaging (MRI) confirmed the final eligibility to participate, and baseline assessments of outcome and safety measures were carried out. Participants were randomly assigned to three groups according to the injectate: MSCs in fibrin glue, fibrin glue/normal saline mixture, and normal saline. The allocation was done via the block randomization method and was performed by the Medical Research Collaborating Center of the institute using block size of three and six.

#### Inclusion and exclusion criteria

We included patients older than 18 years of age with persistent shoulder pain lasting more than 3 months despite conservative treatment, with clinical findings compatible with partial tear of the supraspinatus tendon. To be included, patients were required to have positive empty can tests, decreased elevation/internal rotation of the arm with preserved external rotation in physical examinations, and hypoechoic lesions in the supraspinatus tendon not involving full thickness of the tendon in the ultrasonographic examination later verified by MRI. Those who had steroid injection within 6 weeks prior to initial evaluation; limited range of motion in multiple directions, including external rotation to rule out adhesive capsulitis; full thickness tears of the supraspinatus tendon; symptomatic calcification of the tendon; arthritis; neurogenic atrophy around the shoulder; a history of proximal humeral fracture; or infectious diseases of the affected shoulder were excluded. We additionally excluded those with symptomatic bilateral rotator cuff tears, generalized pain syndrome, ipsilateral radiculopathy, systemic inflammatory diseases, neurological conditions that may affect the assessment, bovine-derived protein allergy, and contraindications to MRI.

Because no previous studies have evaluated the effects of stem cells in human tendinopathy, the target sample size was estimated based on a study of platelet-rich plasma in patients with elbow tendinosis^9^; a size of 24 participants, 8 for each group, was set as the target population.

### Intervention

The intervention group received commercial allogenic adipose tissue-derived adult MSC (Anterogen, Seoul) with fibrin glue^10^ as scaffold. A dual syringe apparatus was adopted. Each syringe was loaded with either stem cells in thrombin, normal saline and thrombin mixture, fibrinogen, or normal saline, according to the allocated group by an independent research assistant. All participants received an intra-lesional injection guided by ultrasonography by a single author in a uniform matter.

Detailed description about the sample size estimation, stem cell preparation, intervention is described in the supplementary methods.

### Follow-up

Outcome measures identical to the baseline assessment were assessed at 6 weeks and again at 3, 6, 12, and 24 months after injection, except for MRI, which was performed at 12 weeks and 12 months after injection. Safety measures and were assessed at 3 days and again at 2, 4, 6, and 12 weeks after injection, and adverse events were appraised at every visit. The participants, care providers, investigators, and outcome assessors were all blinded to the group allocation.

### Study assessments

#### Outcome measures

The severity of pain was assessed by visual analog scale (pain VAS) during activity and resting. Shoulder function was assessed by determination of the American Shoulder and Elbow Surgeons (ASES) score, a questionnaire composed of both physician- and patient-rated components. Patients graded the difficulty performing specific daily activities using a 4-point Likert scale. This method has been shown to be reliable and to yield valid results for rotator cuff disorders^11^. The size of the tear was evaluated by MRI; follow-up images were qualitatively compared with baseline images using a 5-point Likert scale, as follows: markedly increased, slightly increased, stationary, slightly decreased, and markedly decreased. This was used for the statistical analysis, however, for the figure, the 5-point scale was compressed to either “improved”, “no change”, or “aggravated” for visual clarity. Follow-up images were graded by two radiologists (H.S.H, Y.H.J). The assessors were blinded to the intervention and the follow-up period. The inter-rater reliability was good (interclass correlation [standard deviation] = 0.638 [0.348–0.799])^12^.

#### Safety measures

Vital signs; laboratory tests, including complete blood cell counts, erythrocyte sedimentation rate/C-reactive protein, calcium, phosphorus, glucose, blood urea nitrogen/creatinine, uric acid, cholesterol, total protein, albumin, total bilirubin, alkaline phosphatase, glutamic oxaloacetic transaminase/glutamic pyruvic transaminase, sodium, potassium, chloride, and total CO_2_; urine test strips; and urinalysis were evaluated. CD4/CD8 expression levels were evaluated at the baseline and 2 weeks after injection to confirm that there was no prolonged immune reaction after the injection. Adverse events were assessed according to the categorization of Common Terminology Criteria for Adverse Events v4.0.

### Statistical analysis

The primary outcome was the improvement in pain VAS during activity from baseline to 12 weeks after injection. Pain VAS during activity and rest, ASES scores at all time points, and grading of changes in tear size were secondary outcomes. Kruskal–Wallis test was used to compare the primary outcomes between groups, and a mixed effect model was adopted to test whether there were differences in the rate of change among groups in secondary outcomes. The dependent variable was each outcome measure, and the baseline values were included as covariates to adjust for the severity of the condition. The main effects of group and time and the interactions between group and time were the fixed effects of the analyses. Comparisons of the duration and rate of adverse events related to the injection were performed using Kruskal–Wallis tests and chi-square tests, respectively. All analyses were carried out at a 5% significance level.

### Ethical approval and consent to participate

This study involves human participants and was approved by the Institutional review board of Seoul National University Hospital (IRB No. 1404-120-575) and the Korean Ministry of Food and Drug Safety.

## Results

### Participants

**Figure 1:**
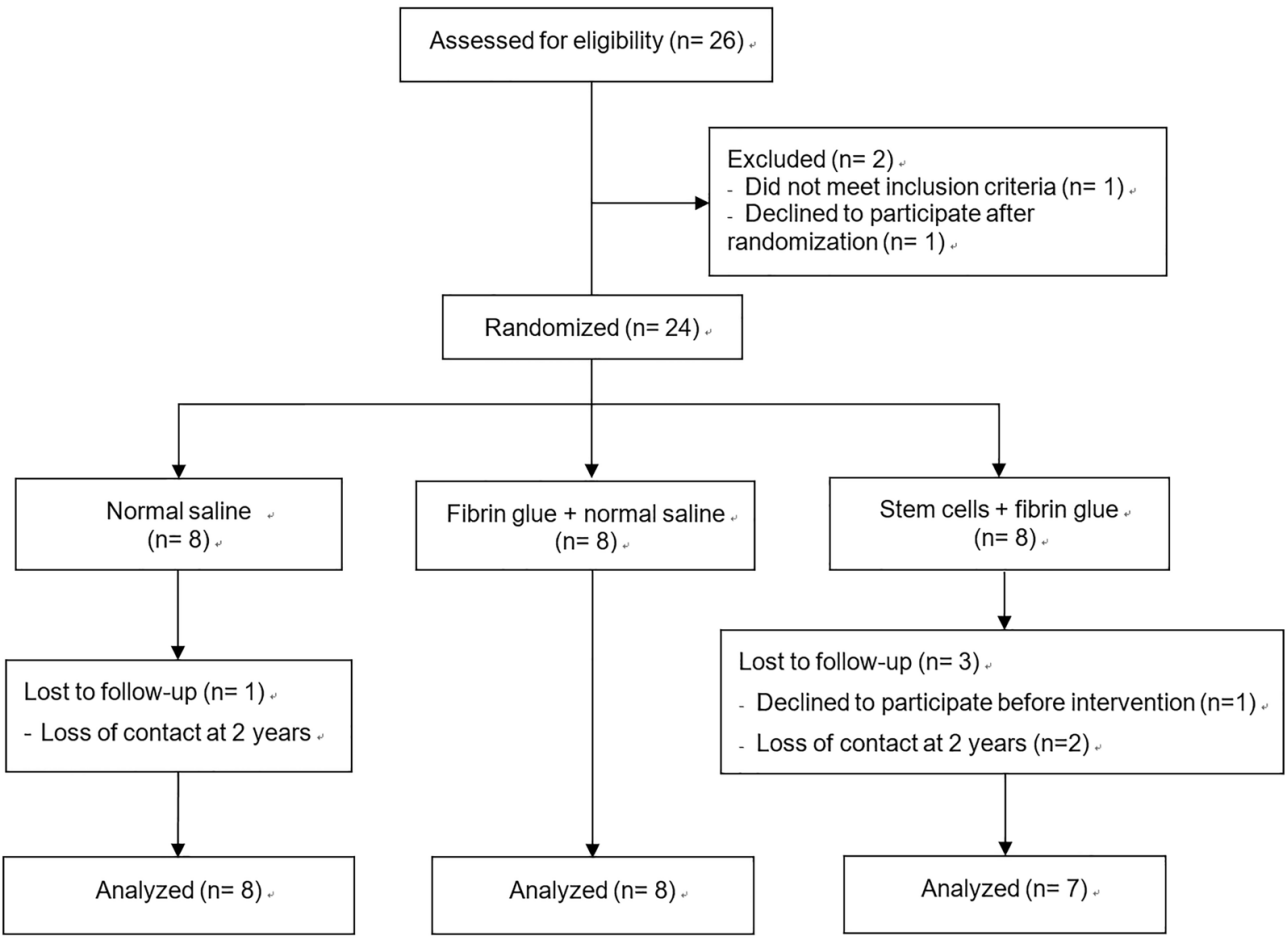
Flow diagram of the study population.

Twenty-six candidates were eligible to participate after the screening. One declined to participate, and another had incompatible MRI findings. The remaining 24 participants were allocated to the three groups. One patient in the stem cell group withdrew after the entire injection procedure was prepared, right before the intervention. At the end of the study, 23 patients were included in the analyses (Fig. 1).

### Outcomes

There were no statistically significant differences in baseline characteristics between groups (Table 1).

**Table 1:** Baseline characteristics by group.

Group by injectate	NS (N = 8)	NS + FG (N = 8)	MSC + FG (N = 7)	*P* value
Sex (Female:Male)	2:6	4:4	4:3	0.41
Age	54.1 ± 9.4	50.4 ± 4.6	61.0 ± 7.8	0.39
Duration (months)	32.6 ± 30.7	24.8 ± 24.2	36.0 ± 24.2	0.55
Dominant extremity affected (No:Yes)	1:7	5:3	2:5	0.10
Active pain	6.6 ± 2.4	4.7 ± 2.6	5.7 ± 1.6	0.20
Resting pain	3.6 ± 1.8	2.4 ± 2.3	3.9 ± 1.8	0.25
ASES	54.7 ± 20.1	64.7 ± 12.8	58.9 ± 12.1	0.46

Values are presented as the mean ± standard deviation or as a number (%). *P* values were obtained using Kruskal–Wallis tests for continuous variables and chi-square tests for categorical variables.

*NS* normal saline, *MCS* mesenchymal stem cell, *FG* fibrin glue, *ASES* American Shoulder and Elbow Surgeons score, *UCLA* University of California, Los Angeles shoulder score, *DASH* Disabilities of Arm, Shoulder and Hand score.

**Figure 2:**
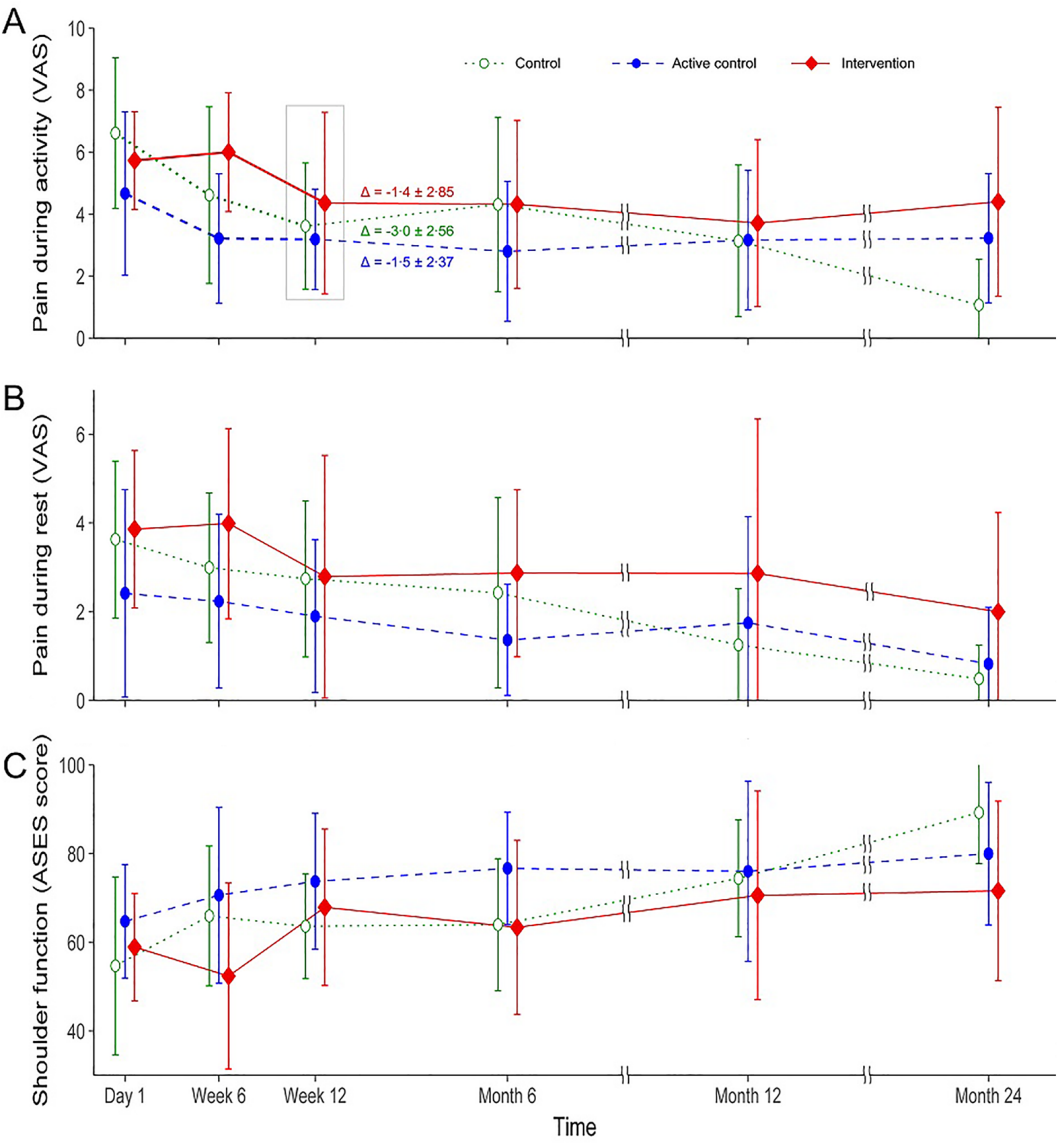
Mean values of pain during activity (**A**) and rest (**B**) assessed by visual analog scale (VAS) and shoulder function (**C**) by American Shoulder and Elbow Surgeons (ASES) score are shown by group and time. The primary outcome, change (Δ) in pain during activity from baseline to 3 months after injection, is noted at the ‘Week 12’ point of the designated group. Whiskers indicate standard deviations.

**Figure 3:**
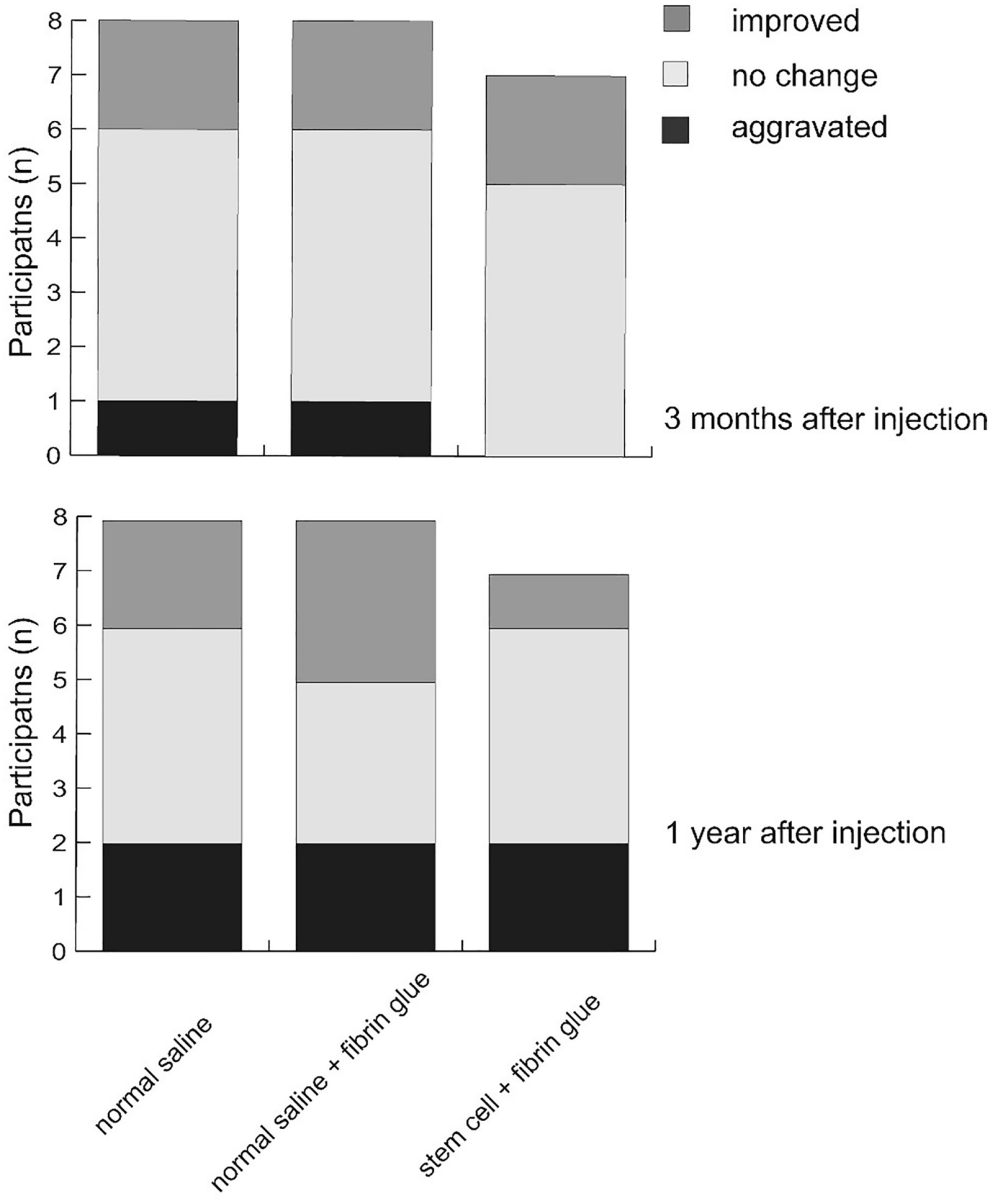
Subjective grading of tear size compared with baseline images.

The mean (standard deviation) changes in pain VAS during activity at 3 months after intervention were − 1.37 (2.85), − 1.48 (2.37), and − 3.0 (2.56) in the intervention, active control, and control groups, respectively. There were no significant differences between groups (*p* = 0.35), although the saline group showed the most improvement (i.e., largest negative net VAS, indicating decreased pain). Pain VAS during activity or at rest, ASES scores, and rates of change in these variables did not differ according to group. However, all clinical variables improved over time (Figs. 2), and MRI grading of tear size showed no significant differences (Fig. 3, Supplementary figure).

### Safety

There were no significant changes in laboratory tests among groups. All participants reported pain at the injection site, which was mild and lasting for 2–3 weeks in the majority. One participant in the active control group had mild pain for 41 days, being alleviated spontaneously. Three patients in each group, 9 in total, had moderate pain that interfered with performing daily activities, of which two from each of the intervention and active control groups, 4 in total, required rescue medication and was given tramadol. Two of those who needed medication improved within two weeks, however, one participant in the intervention group had a subacromial steroid injection at 6 weeks, and one in the active control group had an intra-articular steroid injection at 3 months after injection. The salvage injection had transient effect that did not alter the statistical analysis and no additional treatment was needed. There were no significant differences between groups concerning post-injection pain duration and severity.

## Discussion

This is the first double-blinded, randomized, placebo-controlled trial investigating the effects of intralesional MSC injection in patients with tendinopathy. Although no meaningful safety issues were noted during the trial and subsequent follow-up, MSC injection was not superior to the control treatment regarding improvement of pain and shoulder function in patients with partial thickness tears of the supraspinatus tendon. Changes in lesion size, as assessed by MRI, also did not differ between groups. There were also no differences in the occurrence of adverse events among groups.

### Randomized, controlled clinical trials of stem cell transplantation for tendon defects

When the potential clinical applicability of stem cell treatment was introduced in 2004, many researchers and clinicians were skeptical about illusive promises, such as stem cell treatment for spinal cord injury or stroke, made by flamboyant pioneers of stem cell research. Because there seemed a long way with many difficult, if not impossible, challenges inducing transplanted stem cells to differentiate into functioning neuronal tissues in the damaged central nervous system. However, the positive results of anal fistula trial^4^ lead to an idea that, contrary to highly specialized central nervous tissues, connective tissues may be effectively treated with stem cells. The authors undertook several animal experiments^13^ and a subsequent phase I clinical trial^14^ to acquire positive results. The current phase II clinical trial was carried out with a strong expectation of revealing positive outcomes, extrapolating previous positive results.

In most previous clinical trials that have applied MSCs in tendon disorders, MSCs were implanted as an adjuvant treatment during surgical repair of the tendon. However, recent trials and systematic reviews have disputed the long-term clinical efficacy of repairing nontraumatic rotator cuff tears surgically^15,16^. Thus, along with efforts to improve surgical outcomes in patients with tendinopathies using MSC, similar work should be performed for nonsurgical methods. Studies that have described the nonoperative use of MSCs in patients with tendinopathy^8,14,17^ are all single-arm design which is improper for studies assessing pain as an outcome measure^18^. The current study was the first double-blinded, randomized, controlled clinical trial to investigate the effects of MSCs in tendinopathy.

### Potential pitfalls in study design

Although pioneering work often fails, the negative results of the current study are disappointing considering the promising reports of previous single-arm studies. Successful works are based on trial and error, and we hope that the current study can serve as a foothold to the progress of regenerative medicine in tendinopathy by concisely reviewing the possible causes of the negative results. There were pitfalls in three domains of the study design in the current clinical trial: setting of the inclusion criteria, the control intervention, and estimating the sample size.

The inclusion criteria could not guarantee that the participants all had chronic intractable conditions. Although the mean duration of the symptoms and age in each group did not differ statistically, statistical insignificance cannot assure homogeneity of the participants when the sample size is small. All participants in the group injected with MSCs had a duration of symptoms longer than a year, whereas four patients in the active control group and one patient in the control group had symptoms for less than a year. The uneven distribution of participants with potential for spontaneous recovery may have balanced out the effects of MSCs. Additionally, the wide range of symptom durations within each group might have prevented the study to reveal any possible effect that acts on a certain subgroup with homogenous duration. Similarly, the different age distributions in each group may have contributed to the lack of differences in the results. All but one patient was 60 years old or older in the MSC injection group, whereas only two patient in the control group and no patients in the active control group were of that age. Furthermore, there were no minimum criteria for pain severity. A considerable portion of participants had only mild pain (VAS ≤ 4) at baseline, which left only a small margin for improvement and acted as a ceiling. (Supplementary Data).

In the context of inclusion criteria, we did not exclude volunteers with calcifications when it did not seem to cause any symptoms and many participants had one or multiple small calcifications in the treated tendon. Even a dormant calcification might interfere with the action of the injected MSCs by altering the microenvironment the MSC interacts with. Conversely, the MSC injection might have provoked the existing calcification to progress to a different phase on its natural course which would have ultimately affected the clinical course of the participants. Secondary analysis is warranted, focusing on the outcomes influenced by calcification.

The control groups did not have a purely sham intervention. In the active control group, fibrin glue was injected, and in both control groups, the tendon was pierced. Although fibrin glue has many advantages as a scaffold, it is naturally bioactive and can stimulate cell adhesion and growth^10^. The escalated pain level at 3 days postinjection in both intervention and active control groups (Supplementary Data) is indirect proof of the bioactivity of fibrin glue. Penetrating the tendon is an unnatural stimulus to the tendon, clearly distinct from the causative stimulus of the tear in the supraspinatus tendon. Tendon fenestration itself can have therapeutic effects in chronic tendinopathy^19^. Thus, the therapeutic effects of the control intervention could have diluted the effects of MSCs.

Target sample size is another limitation of the current study. The target sample size was estimated based on a clinical trial that compared platelet-rich plasma and sham injection in epicondylitis of the elbow^9^. This study was the most appropriate reference in the existing literature at the time of conception of the current study because it was the only comparison study that concerned non-surgical application of regenerative medicine in chronic tendinopathy^20^. We are unsure whether sample size estimation based on any previous study would be valid when there is no precedent on nonsurgical stem cell treatment for tendinopathy owing to the fundamental difference between stem cell therapy and other regenerative treatments.

Although the primary outcome measure is not a pitfall of the study, it may be insufficient to assess the effect of the MSCs. Considering the regenerative characteristics of MSC, the intervention might have caused structural changes that did not lead to clinical improvement. We thoroughly reviewed the MRI images both separately and collaboratively to find any meaningful structural change. We also applied the semi-quantitative method adopted in the study by Jo et al.^8^ and reviewed the ultrasonographic examinations that were not depicted in the original protocol. However, we could not observe any difference in regard of structural change between groups.

### Why not in tendinopathy?

Despite all the potential pitfalls discussed above, the most plausible explanation for the current results is that MSCs were not more effective than the control treatments, at least not enough to overcome the potential pitfalls discussed above. Thus, we should speculate as to why no positive results were obtained for patients with tendinopathy, despite positive findings in other disease conditions.

After report of the feasibility of expanding stem cells ex vivo and treating leukemia^21^, stem cells have been applied in various clinical entities, including hematologic disorders, anal fistula in Crohn’s disease^4^, and bone fractures^5^, showed promising results in clinical trials. Blood cells are not attached to the extracellular matrix (ECM) and can function individually to contribute to the system. Thus, in hematologic disorders, stem cells avoid the problem of anoikis^22^ and only need to proliferate and differentiate into functional blood cells. In Crohn’s fistula, the goal of the transplanted MSCs is to produce areolar tissue that simply occupies space and therefore occludes the fistula without the necessity to functionally interact with the surrounding environment. However, in tendinopathy, the stem cells must interact with the ECM and differentiate into tenocytes, which produce and organize the ECM.

In regard of interaction between cells and the ECM, the bone is more similar to the tendon because both tissues function to transmit force via the ECM. However, the bone has a higher metabolic turnover rate and is more vascularized compared with the tendon, which makes the environment more amicable to the introduced stem cells. Additionally, the bone transmits compressive force, which can be conveyed without tissue connection. Thus, in cases of fracture, stem cells can receive adequate physical stimulus once the fracture gap is filled with an appropriate scaffold. In contrast, the tendon transmits tensile force, and in tendinopathy, the fibrous ECM that undertakes the tendon’s function is disrupted. A linear fibrous environment^23^ and tensile loading^24^ are critical in the tenogenic differentiation of MSCs. The lack of both components in tendinopathy is a major impediment to establishing efficient methods for stem cell therapy in patients with tendinopathy. There should be substantial improvement in fundamental issues of stem cell therapy, as discussed below, to overcome these tendon-specific challenges.

### Underlying issues of intralesional injection of stem cells in tendon tears

There are many factors that could be optimized to improve the outcomes of the current intervention. The material, i.e., stem cells, can be either augmented in potency or manipulated for tendon regeneration^25^. The MSCs could be replaced with stem cells of higher hierarchy, such as embryonic stem cells, umbilical cord stem cells, or induced pluripotent stem cells with consideration that higher potency is accompanied by teratogenicity and tumorigenicity issues to be addressed. Alternatively, the stem cells can be harnessed towards tenogenic differentiation^26^ at the cost of somewhat decreased mitotic potential.

The microenvironment of the tendon that interferes the native regenerative process will also impede the effect of the administered MSCs. Preparing the lesion site congenial to the effects of MSC might be necessary. For instance, peppering technique to prompt some intrinsic healing processes^19^ or injecting the MSC with supplementary biological substances^27^ might augment the effect of MSC. Or with multiple MSC injections, separate injections may have priming or boosting effects. There can be limitless administrating methods by combining various routes, scaffolds, volumes, numbers, intervals and adjuvants of injections. Further investigations are required to establish the optimum method.

The scaffold would be of immense significance in the action of MSC. The primary usage of a scaffold is to retain the MSC within the lesion. There were a couple of cases where we could visualize the injectate leaking through a small fissure in the tendon which was not visible before the injection. More cases with such leaks could have occurred that were not captured in sonographic surveillance. It takes a few minutes for fibrinogen to interact with thrombin to form a clot and adhere to the surrounding tissue. Ideally, the scaffold should solidify shortly after administration and at the same time, be malleable enough to conform to the contour of the defect. Additionally, it should be permeable to cytokines and transmit tensile force that stimulate the mechanoreceptors of the MSC to steer it toward tenogenic differentiation.

Whether to mobilize or immobilize the shoulder after the injection could influence the effect of the intervention. In the current trial, participants were educated gentle range of motion exercise and instructed to perform the exercise within tolerable range three times a day. This mobilization would be disadvantageous for holding the injected MSC in site. On the other hand, adequate tensile loading is known to induce tenogenic differentiation of MSC and to refine the extracellular structure to resemble the tendon. Immobilization has detrimental effect on the tendon^28^ and clinical evidence also support functional rehabilitation rather than strict immobilization in tendon injury^29^. Obliging non-painful mobilization after the intervention seems reasonable. However, the intervention is administrating exogenous cells and traditional concepts of mobilizing the tendon might not apply analogously.

### Minor safety issues

All participants had pain at the injection site after the intervention, which should be related to the intervention. Transiently elevated pain can be regarded as an unavoidable cost of the intervention. A single-arm study that adopted a similar MSC injection without scaffolds in the rotator cuff tendon reported no such pain^8^. This may be because the participants underwent arthroscopy, and pain after the intervention was considered natural. The possibility that fibrin glue may trigger a pain-generating process when injected in the shoulder is worth knowing for future investigators. All participants’ pain was manageable with conventional conservative treatment and did not recur during the 2-year follow-up period. The two participants who received additional steroid injections have had intractable shoulder pain lasting for 4 and 5 years each. It is questionable whether these two needed the salvage injection because the pain was similar to the baseline at the injection period. Anyhow, the flared pain caused by the injection process was controlled after a single salvage injection. Ultimately, the post-injection pain was transitory in all subjects. Thus, we consider intratendinous injection of MSC to be safe and believe that pain should not be a constraint to future studies of the application of MSCs in tendons.

#### Our view point on current clinical implications and the future

The current results reporting lesser but nonsignificant clinical effects of MSC injection compared with the control treatment are worth noting. Literature suggesting the potential competence of MSCs in tissue regeneration is rapidly accumulating. Patients with chronic diseases are open to and easily influenced by such information, without proper interpretation. This may result in an inappropriate medical demand for stem cell therapy, and ill-considered applications of MSCs have already spread in clinical practice^30^. Our current findings suggest that in nonsurgical treatment of tendinopathy, the use of MSCs should be confined to research purposes or rigorously selected clinical situations.

The vast discrepancy between preclinical studies that report promising results and insufficient clinical trials cannot be explained solely by the uniqueness of the human body. Clinical trials applying stem cells on tendon lesions has started as early as 2010, according the clinicaltrials.gov, and presumably there would have been more unregistered trials even before. However, the status of most previous trials are unknown, recruiting overdue, terminated or completed without subsequent reports. Not being able to share the details of an unsuccessful clinical trial causes insufficiency in growth of knowledge built up based on trial and error. As discussed above, there are many facets in stem cell therapy for tendinopathy, and literally infinite number of cases in treatment options remains to be investigated. Distribution of all clinical trials, although unsuccessful, followed by a vigorous networked discussion about the results is warranted to develop the optimum method of stem cell treatment for tendinopathy.

## Conclusion

In conclusion, injecting MSCs with fibrin glue into lesions in patients with shoulder pain lasting more than 3 months due to partial tear of the supraspinatus tendon was not superior to injecting fibrin glue with normal saline or normal saline only in terms of pain, shoulder function, and tear size. There were no unmanageable adverse events related to the injection. Further investigations are necessary to establish effective applications of MSCs in the conservative treatment of chronic tendinopathy.

## Supplementary Information

Supplementary Information 1.

Supplementary Information 2.

Supplementary Legends.

Supplementary Information 2.

## Data Availability

The data of the current study is submitted to the journal as supplementary information and will be provided to any researcher when requested to the first author, SW Chun, via e-mail (chun.sewoong@gmail.com).

## Abbreviations

MSC: Mesenchymal stem cell
MRI: Magnetic resonance imaging
pain VAS: Pain assessed by visual analog scale
ASES: American Shoulder and Elbow Surgeons
CD: Cluster of differentiation
ECM: Extracellular matrix
