# Supplementary figures and images for: A randomized controlled trial of stem cell injection for tendon tear
Source: Sci Rep. 2022 Jan 17;12:818. doi: 10.1038/s41598-021-04656-z (PMC8764049; doi:10.1038/s41598-021-04656-z)

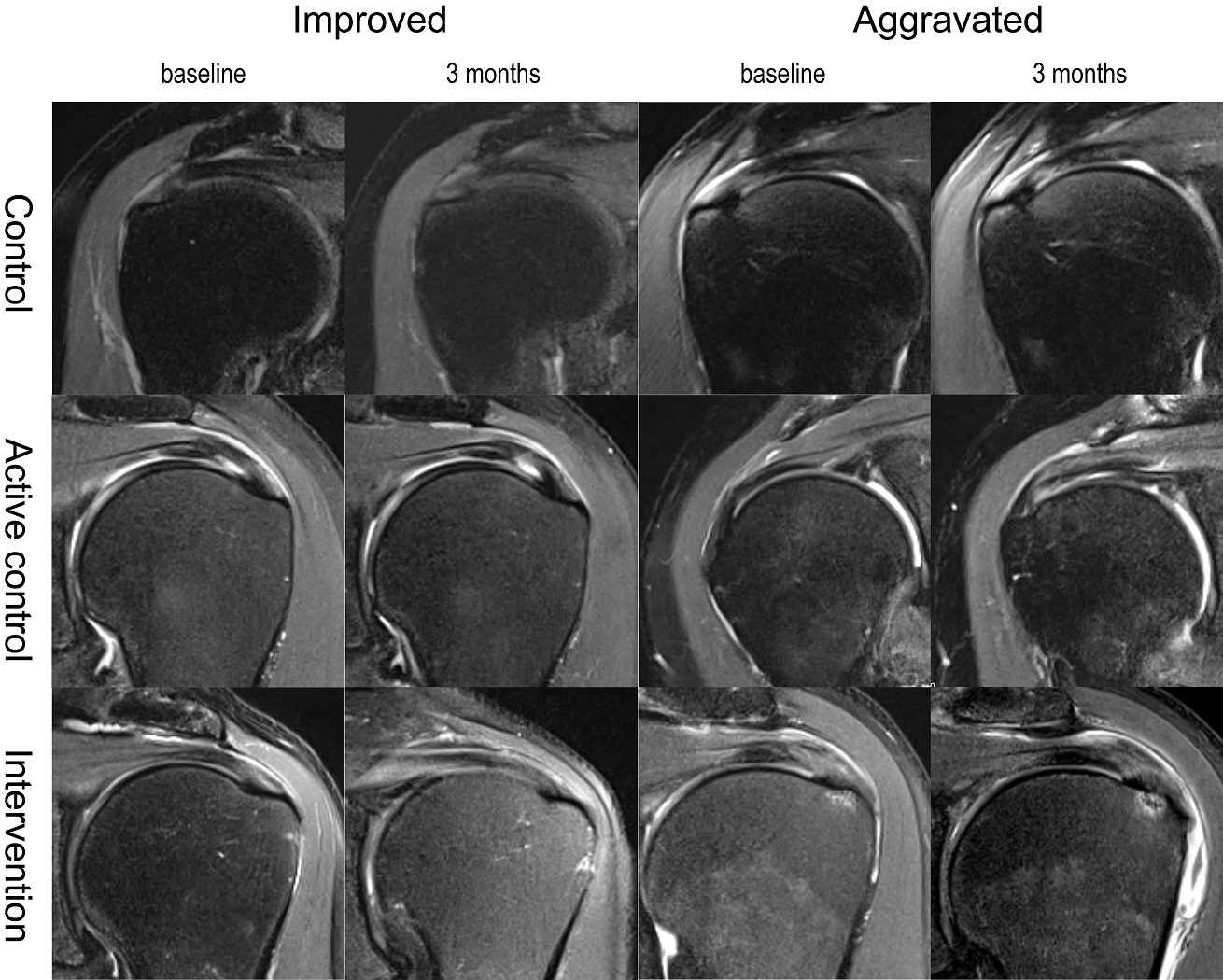

Supplement: Supplementary file 2 — Supplementary Information 2. [file 41598_2021_4656_MOESM2_ESM.jpg]
