# Supplementary material for: A randomized controlled trial of stem cell injection for tendon tear
Source: Sci Rep. 2022 Jan 17;12:818. doi: 10.1038/s41598-021-04656-z (PMC8764049; doi:10.1038/s41598-021-04656-z)
Supplement: Supplementary file 3 — Supplementary Legends. [file 41598_2021_4656_MOESM3_ESM.docx]

Supplementary figure. Images at baseline and 3 months after the intervention of those with most improvement/deterioration in the primary outcome (change in pain during activity from baseline to 3 months after intervention).
