# Supplementary material for: A randomized controlled trial of stem cell injection for tendon tear
Source: Sci Rep. 2022 Jan 17;12:818. doi: 10.1038/s41598-021-04656-z (PMC8764049; doi:10.1038/s41598-021-04656-z)
Supplement: Supplementary file 4 — Supplementary Information 2. [file 41598_2021_4656_MOESM4_ESM.docx]

***Sample size estimation***


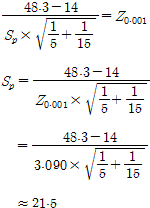
Because no previous studies have evaluated the effects of stem cells in human tendinopathy, the target sample size was estimated based on a study of platelet-rich plasma in patients with elbow tendinosis.^1^ Each group had equal number of subjects and the level of significance was set as α = 0.05 while the power of test was 80%. In the previous study,^1^ the intervention group (n = 15) had 48.3 points improvement in pain assessed by visual analog scale (VAS) and the control group (n = 5) had 14 points improvement. The two groups had significant difference (p = 0.001) in unpaired t-test. Based on the aforementioned information the pooled standard was calculated as follows;

The estimated target number per group was calculated using PASS (PASS Software (Power Analysis and sample size software : <http://www.ncss.com)>). Considering a drop-out of 10%, 8 subjects in each group, a total of 24 participants were recruited.

***Stem cell preparation***

Human subcutaneous adipose tissue was harvested from a healthy female donor after obtaining informed consent. As the batch size of ASC bank from one donor is at least more than 50, the ASCs at passage 3~4 obtained was used for this clinical trial. The lipoaspirate was immersed and gently shaken in phosphate-buffered saline containing 1% bovine serum albumin and 0.025% collagenase type 1 for 80 min at 37°C. The stromal vascular fraction was extracted and cultured in Dulbecco’s modified Eagle’s medium (DMEM; Invitrogen, Gaithersburg, MD http://www.thermofisher.com) supplemented with 10% fetal bovine serum and 1 ng/mL basic fibroblast growth factor. The cultured cells (allogenic adipose-derived adult MSCs) were harvested via trypsinization, suspended in DMEM at a concentration of 3.0 × 10^7^ cells/mL, and packaged into disposable vials (Anterogen, Seoul, Korea <http://anterogen.com/>). The products were manufactured according to the Good Manufacturing Practices of the Korean Ministry of Food and Drug Safety. The manufactured stem cells were characterized by their cluster of differentiation (CD) cell surface markers. They expressed stromal cell-associated markers (CD10, CD13, CD29, and CD 90), but were negative for hematopoietic/bone marrow-derived stem cell markers (CD34, CD45, and Stro-1). Assessments of cell appearance, viability, identification, purity, content, and potency showed that the cells composed the lot release testing. The minimum criteria for release were as follows: 80% viability and less than 1% of cells expressing CD45. Potency and genomic stability were assessed by viable cell counting and karyotyping. No evidence of tumor formation was reported after repeated administration of cell to immune-compromised nude mice. Sterility was guaranteed after screening for adventitious agents, mycoplasma, and other bacteria, fungi, viruses, and endotoxins 3 days prior to packaging and sample testing of the final product to comply with the “Guidance on specifications and test methods for cell therapy products” from the Korean Food and Drug Administration.

A dual 1-mL syringe injection system (Greenplast kit; Greencross, Seoul, Korea <http://globalgreencross.com/>) with a 22-guage spinal needle was adopted. The stem cell-injected group received 0.5 mL of a mixture of 1.0 × 10^7^ MSCs in 0.2mL cell solvent and 0.3mL thrombin and 0.5 mL of fibrinogen. The number of MSCs was selected based on a previous study^4^ that proved the safety of intra-tendinous injection of the same MSC product. The thrombin was added to the MSC mixture such that after inoculation into the lesion, fibrinogen would be converted into an insoluble fibrin matrix (fibrin glue), an excellent three-dimensional scaffold^5^ and could contain MSCs within the lesion. In the active control group, we substituted normal saline for MSC suspension, and the control group was injected with normal saline in both syringes.

***Ultrasound-guided intralesional injection***

Intralesional injection of 1 mL was performed under ultrasonographic guidance by the senior author (C.S.G.) for all participants. Patients were positioned in the supine position, with the shoulder rotated internally to expose the supraspinatus tendon as much as possible under the acromion, and sonographic scanning was performed to visualize the whole tendon by moving the probe from the long biceps tendon to the infraspinatus tendon in the longitudinal view and from the footprint of the tendon to the acromion in the transverse view. After scanning the whole length and width of the tendon, the probe was fixed at a position where the partial tear, a sonographic defect with low echogenicity with loss of fibrillar patterns, appeared the largest in both viewing planes. After confirming that the sonographic tendon defect coincided with one in the baseline MRI, redundant coupling gel around the probe was cleaned, and the skin area where the needle puncture was anticipated was thoroughly scrubbed repeated with betadine-soaked cotton balls. Without moving the probe or the patient’s arm, the needle was introduced into the tendon by piercing the skin at least 1 cm from the end of the probe to maintain strict aseptic conditions. The direction of needle advancement was in plane with the sonographic view such that the whole needle tip and shaft in the tendon could be visualized at any time during the injection procedure. When the needle tip reached the center of the tendon defect, the plunger of the dual syringe was pushed slowly but steadily. Because the injectionist was looking at the ultrasound monitor to secure the needle tip at the center of the defect, there was an attending nurse to read volume markings on the syringe such that the infusion was stopped after injection of 1 mL. Aseptic dressing was applied after the injection and the participant was followed up 3 h later for physical signs of inflammation, such as severe pain, swelling, and heating sense in the injected shoulder, and by ultrasonography to check for the occurrence of edema or effusion and to confirm that the injectate remained at the injected site.

**References**

1. Mishra A, Pavelko T. Treatment of chronic elbow tendinosis with buffered platelet-rich plasma. The American journal of sports medicine 2006;34:1774-8.

2. Kocher MS, Horan MP, Briggs KK, Richardson TR, O'Holleran J, Hawkins RJ. Reliability, validity, and responsiveness of the American Shoulder and Elbow Surgeons subjective shoulder scale in patients with shoulder instability, rotator cuff disease, and glenohumeral arthritis. The Journal of bone and joint surgery American volume 2005;87:2006-11.

3. Cicchetti DV. Multiple comparison methods: establishing guidelines for their valid application in neuropsychological research. Journal of clinical and experimental neuropsychology 1994;16:155-61.

4. Lee SY, Kim W, Lim C, Chung SG: Treatment of Lateral Epicondylosis by Using Allogeneic Adipose-Derived Mesenchymal Stem Cells: A Pilot Study. Stem cells (Dayton, Ohio) 2015, 33(10):2995-3005.

5. Wu X, Ren J, Li J: Fibrin glue as the cell-delivery vehicle for mesenchymal stromal cells in regenerative medicine. Cytotherapy 2012, 14(5):555-562.
